# John Hunter

**Published:** 1860-12

**Authors:** 


*John Hunter.*—The Council of the Royal College of Surgeons have caused a beautiful memorial tablet to be placed over the site of the grave of Hunter, now resting in Westminster Abbey, with the following inscription:—

"Beneath are deposited the remains of John Hunter. Born in Long Calderwood, Lanarkshire, N. B., on the 13th of February, 1728. Died in London on the 16th of October, 1793. His remains were removed from the Church of St. Martin's-in-the-fields to this Abbey, on the 28th of March, 1859. The Royal College of Surgeons of England have placed this tablet over the grave of Hunter, to record their admiration of his genius, as a gifted interpreter of the divine power and wisdom at work in the laws of organic life, and their grateful veneration for his services to mankind as the founder of scientific surgery."

This inscription is deeply cut in brass, of a Gothic design, inlaid in a slab of polished red granite, and presents that chaste and elegant appearance for which the Messrs. Hardman, of Birmingham, who executed the work, are so distinguished. Mr. Weekes, the eminent sculptor, is progressing favorably with the model of the statue, which is to be of marble, and to be placed in the Hunterian Museum, thus to be comparatively buried. Why was not a public site chosen for it? Mr. South, the president of the college, still continues to receive subscriptions toward the foundation of a scholarship (after payment for the statue), in order to perpetuate still more the immortal genius of Hunter. Our transatlantic brethren have already sent a handsome sum to Mr. South, as a first installment towards this desirable project.

